# Refractive Outcomes in Keratoconus Patients Following Toric Lens Implantation: A Systematic Review and Single-Group Meta-Analysis

**DOI:** 10.3390/life15091362

**Published:** 2025-08-27

**Authors:** Tun Giap Tan, Kieran O’Kane, Harry W. Roberts

**Affiliations:** 1 Torbay Hospital, Torbay and South Devon NHS Foundation Trust, Torquay TQ2 7AA, UK; tungiap.tan@nhs.net; 2 Royal Eye Infirmary, University Hospitals Plymouth NHS Trust, Plymouth PL6 5ZF, UK; kieran.okane@nhs.net; 3 Faculty of Health and Life Sciences, University of Exeter Medical School, Exeter EX1 2LU, UK; 4 West of England Eye Unit, Royal Devon University Healthcare NHS Foundation Trust, Exeter EX2 5DW, UK

**Keywords:** keratoconus, toric intraocular lens, astigmatism

## Abstract

This systematic review and meta-analysis evaluated refractive outcomes, particularly astigmatic correction, in keratoconus following toric intraocular lens (tIOL) implantation. A systematic search identified eligible studies reporting pre- and postoperative refractive cylinder, spherical equivalent (SE), uncorrected distance visual acuity (UDVA), and corrected distance visual acuity (CDVA). Eight studies, comprising 135 eyes, were included. Outcomes were pooled using a random-effects model with restricted maximum likelihood as the estimator for tau^2^. Methodological quality was assessed using the MINORS tool for non-comparative studies and the JBI checklist for case series. Postoperative refractive cylinder and SE improved by 2.28 dioptres (95% CI, 1.60–2.96) and 4.17 dioptres (95% CI, 2.32–6.01), respectively. UDVA and CDVA also improved substantially, with pooled gains of 0.87 logMAR (95% CI, 0.71–1.03) and 0.19 logMAR (95% CI, 0.12–0.26), respectively. Most tIOL rotations did not exceed 10 degrees, with only one case requiring realignment surgery. Complications were infrequent and mostly minor. tIOL implantation is effective in reducing astigmatism and improving vision in stable keratoconus patients. However, limitations in vector analysis and methodology heterogeneity underscore the need for standardised reporting to optimise outcomes.

## 1. Introduction

Keratoconus (KC) is a bilateral, asymmetric, progressive ectatic condition characterised by central or paracentral corneal stromal thinning. The resultant apical protrusion of the cornea leads to decreased visual acuity due to induced myopia, astigmatism, and higher-order aberrations (HOAs) [1,2,3,4,5]. HOAs are complex refractive errors arising from imperfect focusing of light through the optical system, which cannot be corrected with conventional spherocylindrical lenses [6].

Toric intraocular lens (tIOL) implantation is an established option to neutralise regular corneal astigmatism during cataracts, refractive lens exchange (RLE), phakic IOL, or supplementary IOL surgery, improving uncorrected distance visual acuity (UDVA) [7,8,9,10,11,12,13]. tIOLs have been reported as a feasible option for eyes with stable KC and repeatable astigmatism, with several case reports demonstrating promising results in select patients [14,15,16]. A recent meta-analysis examined spherical equivalent (SE) outcomes after tIOL implantation in KC; however, it did not evaluate astigmatic outcomes, which are of particular interest when implanting such prostheses [17]. Predominantly coma-type HOAs in KC affect manifest astigmatism [18,19,20,21,22,23], and optical quality is in turn influenced by complex interactions between coma and astigmatism [24,25,26]. These factors create practical uncertainties in selecting the cylinder magnitude and the axis for tIOL implantation in irregular corneas.

To our knowledge, this is the first systematic review with a meta-analysis reporting the astigmatic outcomes of tIOLs in KC. This review aims to examine the available evidence and investigate refractive outcomes in terms of lower-order wavefront aberrations in KC patients following monofocal tIOL implantation.

## 2. Methods

### 2.1. Search Strategy

This systematic review with a meta-analysis was conducted in accordance with the Cochrane Handbook and reported using the PRISMA 2020 guidelines [27]. A systematic literature search was performed on electronic databases, including PubMed/MEDLINE, Embase Ovid, and Google Scholar, using relevant keywords, phrases, and medical subject heading terms. The full search strategy is provided in the Supplementary Material. Clinical trial registries, including the Cochrane Central Register of Controlled Trials (CENTRAL) and the ISRCTN registry, were also searched. The literature from inception until 27 April 2023 was reviewed, with an updated search performed on 31 December 2024. Keywords used in the literature search included the following: (keratoconus OR keratoconic) AND (phacoemulsification OR cataract surgery OR cataract operation OR intraocular lens). The study protocol was registered in the International Prospective Register of Systematic Reviews (PROSPERO 2023, https://www.crd.york.ac.uk/prospero/, accessed on 28 April 2023: registration number, CRD42023422070).

### 2.2. Study Eligibility Criteria

All prospective and retrospective studies meeting the following inclusion criteria were considered: (1) articles published in English; (2) studies of the following designs: randomised controlled trial, case–control, cohort, or case series; (3) patients aged 18 or above with stable, non-progressing KC, including those with prior corneal cross-linking, who received a monofocal toric lens implant via refractive lens exchange or cataract extraction by phacoemulsification; (4) studies reporting outcomes relevant to this review. Exclusion criteria were (1) patients with stage 4 keratoconus according to the Krumeich classification or central corneal scarring; (2) patients with other ocular comorbidities such as glaucoma, maculopathy, retinopathy, or ocular surface inflammation; (3) patients with previous refractive or corneal surgery, excluding corneal cross-linking; (4) combined procedures such as phacoemulsification with keratoplasty, intrastromal corneal ring segments, or corneal cross-linking therapy; (5) conference abstracts or posters.

### 2.3. Screening and Data Extraction

Duplicate articles from the database search were removed using the web version of reference management software EndNote. The remaining studies were screened independently by two authors (T.G.T., K.O.K.) using the systematic review screening software Rayyan QCRI. Titles and abstracts were initially screened, followed by full-text review for study inclusion. Disagreements were resolved through consultation with a third author (H.W.R.).

Outcome measures were independently extracted by two authors (T.G.T., K.O.K.) using a pre-specified template to capture study characteristics (author, publication year, country, study design), sample characteristics (size, sex, age at presentation), baseline parameters (spherical equivalent [SE], refractive cylinder, uncorrected distance visual acuity [UDVA], and corrected distance visual acuity [CDVA]), and outcomes (SE, refractive cylinder, UDVA, CDVA, complications, intraocular lens rotation, and length of follow-up). Extracted data were then discussed and double-checked to ensure completeness and accuracy. Two attempts via email were made to contact the corresponding author of any study that met the inclusion criteria but had missing data. Studies with insufficient responses were excluded to preserve statistical rigour.

### 2.4. Quality Assessment

Quality assessment of non-comparative studies was performed using the Methodological Index for Non-Randomized Studies (MINORS) tool, which assesses studies across domains such as patient selection, outcome measures, outcome reporting, and follow-up duration [28]. Each study was assigned a total score out of 16 based on an 8-item checklist, with scores of ≤8 indicating poor methodological quality, 9–14 indicating moderate quality, and ≥15 indicating good quality. All non-comparative studies in this review were deemed to be of moderate methodological quality (see Supplementary Table S1). The methodological quality of case series was assessed using the JBI critical appraisal checklist for case series, which comprises 10 questions addressing factors such as complete patient inclusion, outcome reporting, and the appropriateness of statistical analysis (see Supplementary Table S2) [29]. Exclusion of studies based on quality assessment was not attempted due to the limited available literature at the time of this review.

### 2.5. Outcome Measures and Statistical Analysis

The primary outcomes were the mean difference in refractive cylinder, SE, UDVA, and CDVA following tIOL implantation. These were calculated using preoperative and postoperative means and standard deviations (SDs) reported in the included studies. Given the anticipated between-study heterogeneity, pooled outcomes were calculated using a random-effects model with restricted maximum likelihood (REML) [30] as the estimator for tau^2^. Secondary outcomes included postoperative tIOL rotation and complications. Publication bias was planned to be assessed via funnel plot asymmetry using Egger’s test. However, this was not performed due to the small number of studies, which would render the test underpowered to distinguish chance from true asymmetry [31]. All analyses were performed using the “meta-for” and “effectsize” packages in R (version 4.4.1).

## 3. Results

### 3.1. The Literature Search

A total of 941 records were identified from the initial and rerun searches of electronic databases, including PubMed and Embase. After removing 353 duplicates, 588 records remained for title and abstract screening, of which 42 were selected for full-text evaluation. Forty-one of these full-text reports were successfully retrieved. Following full-text screening, eight original studies were included in this review (Table 1) [32,33,34,35,36,37,38,39].

### 3.2. Primary Outcomes

**Figure 1:**
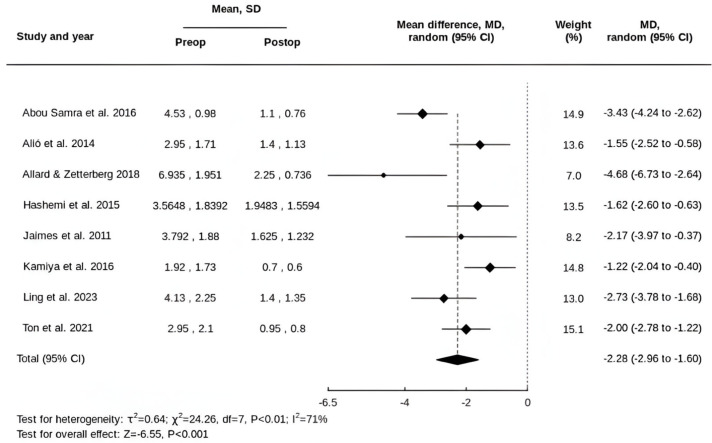
Forest plot of the MD in refractive cylinder following tIOL implantation [32,33,34,35,36,37,38,39].

A total of 135 eyes from 102 patients were included across the eight studies. One patient (one eye) in the retrospective study by Jaimes et al. had a dense cataract, preventing measurement of preoperative refraction and visual acuity, and was therefore excluded from the meta-analysis [36]. Preoperatively, the mean refractive cylinder was 3.38 ± 2.11 dioptres (D), while the mean residual refractive cylinder postoperatively was 1.30 ± 1.17 D. The pooled mean difference (MD) in the refractive cylinder was a reduction of 2.28 D (95% CI, 1.60 to 2.96) (Figure 1).

**Figure 2:**
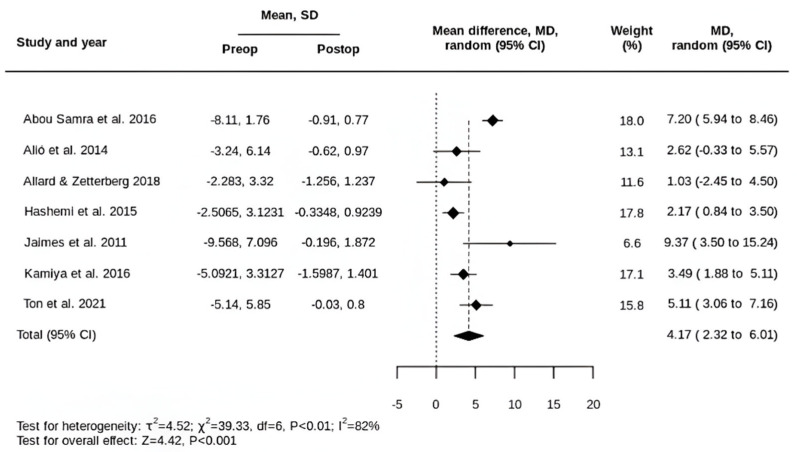
Forest plot of the MD in SE following tIOL implantation [32,33,34,35,36,37,39].

For SE, the preoperative mean was −4.67 ± 5.08 D, and the postoperative mean was −0.58 ± 1.17 D. The pooled MD in SE was a reduction of 4.17 D (95% CI, 2.32 to 6.01 D) (Figure 2).

**Figure 3:**
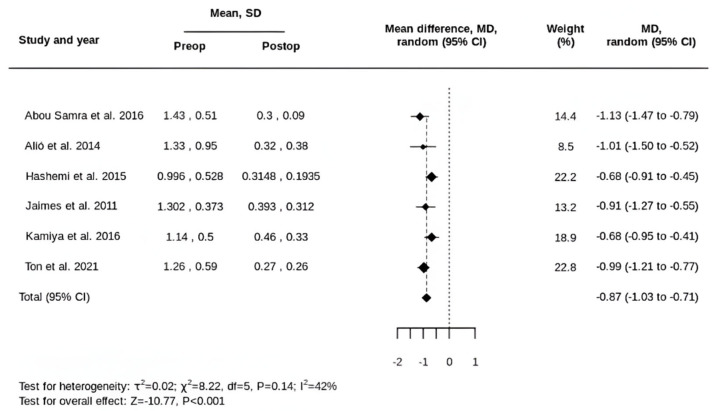
Forest plot of the MD in UDVA following tIOL implantation [32,33,34,35,36,39].

In terms of UDVA, six of the eight included studies provided sufficient data, comprising 107 eyes from 67 patients. The preoperative mean was 1.21 ± 0.62 logMAR, and the postoperative mean was 0.33 ± 0.28 logMAR. The pooled MD in UDVA following tIOL implantation was a gain of 0.87 logMAR (95% CI, 0.71 to 1.03) (Figure 3).

**Figure 4:**
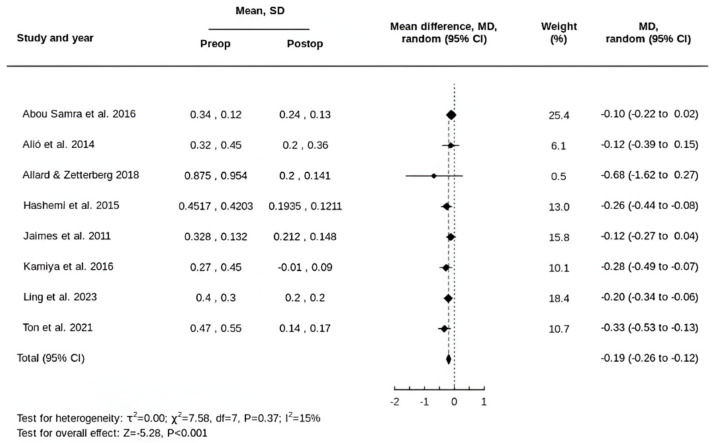
Forest plot of the MD in CDVA following tIOL implantation [32,33,34,35,36,37,38,39].

Following tIOL implantation, the mean CDVA improved from 0.40 ± 0.45 logMAR preoperatively to 0.16 ± 0.20 logMAR postoperatively, corresponding to a pooled mean difference of 0.19 logMAR (95% CI, 0.12 to 0.26) (Figure 4).

### 3.3. Secondary Outcomes

Only four of the eight included studies reported postoperative tIOL rotation. Allard & Zetterberg reported 8 degrees of tIOL rotation in one eye, the most significant in their case series [34]. Hashemi et al. reported mean tIOL rotations of 2.50 ± 1.18 degrees (range, 1–5), 2.50 ± 1.51 degrees (range, 0–6), and 2.67 ± 1.15 degrees (range, 2–6) in the mild, moderate, and severe keratoconus groups, respectively [35]. Jaimes et al. documented three cases requiring tIOL realignment: one due to haptic luxation, one due to tIOL misalignment, and one due to tIOL rotation [36]. Kamiya et al. reported a mean tIOL rotation of 5.2 ± 2.6 degrees (range, 1–10) [37].

Amongst the eight studies, three reported postoperative complications. Both Allard & Zetterberg and Kamiya et al. noted a single case of postoperative cystoid macular oedema (CMO) in their cohorts [34,37]. Jaimes et al. reported two cases of wound leak, with one requiring a re-suturing and the other managed with a bandage contact lens [36].

## 4. Discussion

Stable KC is a prerequisite for tIOL implantation. Only three of the included studies specified criteria for defining non-progression of KC preoperatively. These criteria were inconsistent and were based on a combination of stability of keratometry, refraction, and pachymetry over a 6- to 12-month period [33,35,36]. In the study by Abou Samra et al., the cohort had progressive KC and underwent a two-stage procedure, consisting of corneal cross-linking (CXL) followed by tIOL implantation six months later [32]. Although the refractive outcomes in this study appeared favourable, the sample size was too small to draw generalisable conclusions.

All the studies in this meta-analysis reported a reduction in mean SE following tIOL implantation. However, this gives no indication of the effectiveness of astigmatism correction. In this review, our primary area of interest was cylindrical outcomes, as SE outcomes have been described in previous studies [17,40,41]. All eight included studies reported a reduction in mean refractive astigmatism in keratoconic eyes following tIOL implantation.

Preoperatively, the mean astigmatism was 3.38 ± 2.11 dioptres (D), which is relatively low for a cohort of keratoconic eyes. Some included studies excluded patients with more severe KC based on the Amsler–Krumeich classification system, which incorporates keratometry, optical pachymetry, refraction and biomicroscopy [42,43]. In studies that included patients with more severe KC, the cohort was skewed towards mild or moderate cases. An important source of heterogeneity in this review arises from the refraction method. Five out of eight studies reported manifest refraction, with Abou Samra et al. including at least one cycloplegic refraction during follow-up, without further specification [32,35,36,37,38]. In the series by Allard & Zetterberg, two of the four patients were assessed with autorefraction, and two studies did not specify the refraction method used [34,36,39]. A prior study by Soeters et al., comprising 90 keratoconic eyes of 61 patients, concluded that autorefraction is unreliable in KC, with greater discrepancies observed in steeper corneas [44].

It is worth noting that two cases reported by Kamiya et al. had high residual astigmatism, partly due to the unavailability of higher-powered tIOLs in the study country at the time [37]. Additionally, a frequently cited study by Nanavaty et al. was not included in this review because one of the twelve eyes had a prior Intacs corneal implant, and there was insufficient information to separate its data from the rest. This study similarly reported a significant reduction in astigmatism magnitude, from 3.00 ± 1.00 D to 0.70 ± 0.80 D [45]. Although studies show that astigmatism less than 0.5 D does not degrade visual acuity [46,47], reporting only postoperative mean values does not distinguish patients who achieve <0.5 D of residual astigmatism from those who do not. Therefore, Holladay et al. recommended reporting the percentages of patients within specific residual astigmatism intervals [48]. Some studies in this review reported only the postoperative mean astigmatism.

Astigmatism is defined by both magnitude and axis. Evaluating postoperative residual astigmatism in terms of its magnitude is useful, given its correlation with UDVA regardless of axis change [46,47]. However, assessing change in astigmatism magnitude alone is insufficient to determine the effectiveness of an astigmatism correction. Alió et al. reported a vector analysis using the Alpins method in their study [33]. Kamiya et al. presented postoperative refractive outcomes using power vector analysis as described by Thibos and Horner [37]. Power vectors represent spherocylindrical refraction geometrically in three independent dioptric components, permitting a more detailed description of postoperative refractive changes [49]. Other studies reported a simple subtraction or scalar analysis, comparing preoperative and postoperative astigmatism in terms of magnitude alone.

The Alpins method evaluates the effectiveness of astigmatism treatment by examining the relationships between three fundamental vectors, namely, target-induced astigmatism (TIA), surgically induced astigmatism (SIA), and difference vector (DV) [49,50]. TIA represents the intended astigmatic treatment of an intervention, in both magnitude and axis. SIA is the vectorial difference between preoperative and postoperative astigmatism, which can be corneal or refractive. DV is the residual astigmatic correction required to achieve the TIA. DV is an absolute measure of success and equals zero when SIA exactly matches TIA. The correction index (CI), defined as the ratio of SIA to TIA, indicates overcorrection when greater than one and undercorrection when less than one [50,51]. Apart from Alió et al. [33], the studies in this review did not report TIA, precluding vector analysis. Since stock tIOLs are available only in fixed dioptric steps, TIA cannot simply be assumed to achieve a postoperative zero-dioptre target. Without vector analysis using TIA as a reference, residual astigmatism alone provides insufficient information to determine under- or overcorrection.

In the Alpins method, aggregate analysis of multiple eyes using double-angle vector diagrams (DAVDs) permits calculation of systematic errors, such as undercorrection, overcorrection, or misalignment, which are reported as geometric means [50]. This visualisation can guide adjustments to improve postoperative refractive outcomes. Of the eight included studies, only Ling et al. reported preoperative and postoperative astigmatism using a DAVD [38]. Aggregate data can also be presented using surgical polar vector diagrams, which halve the astigmatism meridian and axis values used in DAVD calculations [50]. To standardise outcome reporting, *The Journal of Refractive Surgery* (JRS) provides standard graphs illustrating the proportions of eyes with under- or overcorrection of astigmatism in both magnitude and axis [52]. None of the studies in this review adhered to the JRS standard for reporting astigmatic outcomes.

The cornea contributes approximately three quarters of the eye’s refractive power [1], making accurate keratometry essential for optimising preoperative tIOL calculations. In KC, the orthogonally asymmetric cornea with a displaced optical centre produces coma-like aberrations [18,19,20,21,22], which clinically contribute to manifest astigmatism [23]. Keratometry can be challenging in KC due to corneal irregularities, and associated corneal HOAs may explain the lower predictability of postoperative refractive outcomes in severe KC [17,40,41,53,54]. The location of corneal ectasia also affects refractive predictability, with more central ectasias having a greater impact on keratometry accuracy along the visual axis [33]. Multiple corneal imaging devices are currently available, including Placido-based systems and corneal tomography techniques such as scanning slit, Scheimpflug, and optical coherence tomography (OCT)-based devices. Placido-based devices provide corneal topography for mapping the anterior corneal surface, whereas corneal tomography techniques generate three-dimensional maps, including posterior elevation, which is particularly relevant in KC [55,56]. Studies report varying levels of inter-device agreement, with lower device repeatability observed in more advanced KC [18,57,58].

Most studies in this review employed multiple devices to derive keratometric data, and some applied different methods for different patients, representing another important source of heterogeneity. None of the studies used newer devices such as the Sirius (Costruzione Strumenti Oftalmici, CSO), which combines a rotating Scheimpflug camera and a small-angle Placido-disk topographer, or the MS-39 (CSO), which integrates an anterior-segment OCT with a Placido-disk topographer. Both the Sirius and MS-39 had demonstrated good repeatability of keratometry in keratoconic eyes [59,60].

A linear relation between refractive astigmatism (RA) and keratometric astigmatism (KA) was first proposed by Émile Javal in 1890 and subsequently refined by others [61,62,63]. Historically, the disparity between the two astigmatic values was attributed to lenticular astigmatism (LA) [64]. However, studies have shown that this discrepancy persists even in pseudophakic eyes [65,66]. Koch et al. suggested that posterior corneal astigmatism (PCA) accounts for the difference between RA and KA [64,67]. Conventional or simulated keratometry (Sim-K) estimates KA using the measured anterior corneal curvature and a standard keratometric index (1.3375), assuming a fixed ratio between anterior and posterior corneal curvatures [22,68]. In KC, this ratio is not constant, leading to inaccurate postoperative refractive outcomes [53,55,69,70,71]. Two case series, comprising 137 and 161 eyes with KC, respectively, reported that PCA accounts for approximately one fifth of total corneal astigmatism (TCA), highlighting its clinical relevance [72,73].

Incorporation of PCA into tIOL calculations was first introduced using Koch’s Baylor nomogram [64]. Subsequently, Holladay and George devised a formula accounting for total surgically induced astigmatism (SIA) to improve postoperative astigmatism outcomes [74]. Other methods, including the Barrett Toric Calculator, Kane Toric formula, and Naeser–Savini formula, incorporate all three vectors (keratometry, PCA, and SIA) to determine tIOL toricity and axis [75,76,77]. Application of the Abulafia–Koch (AK) regression formula has been reported to further improve postoperative astigmatic outcomes by enabling more accurate TCA estimation [78]. The Barrett Toric Calculator can use either predicted or measured PCA, with studies showing comparable postoperative results between the two, as well as with the Kane Toric formula and AK regression formula [79,80]. To date, no study has evaluated the predictability of astigmatic outcomes in KC comparing the different tIOL formulae.

Apart from Alió et al., all included studies used tIOL calculators provided by the respective manufacturers, with the AcrySof Toric IOL calculator being the most frequently cited. It was unclear whether the Barrett Toric or the Holladay Total SIA Toric Calculator within the AcySof Toric IOL calculator was used in these studies. Additionally, the studies did not specify whether predicted or measured PCA was used in preoperative calculations. Intraoperative aberrometry, another method of tIOL calculation, was not utilised in any of the studies [81].

Another important vector in the preoperative analysis of tIOL is SIA_Cornea_, which represents the change in corneal astigmatism resulting from corneal incisions [82]. Abulafia et al. recommended using the term SIA_Cornea_ to differentiate it from the astigmatic change induced by a tIOL, SIA_IOL_, or by the surgery as a whole, SIA_Total_ [83]. None of the included studies specified their method of calculating SIA_Cornea_, a variable influenced by multiple factors including corneal incision location [83]. The varied corneal incision sizes and locations across studies represent important sources of heterogeneity in this review. Corneal incision sizes ranged from 1.0 mm to 3.2 mm. Febbraro et al. reported that 3.2 mm incisions induce greater astigmatic effects than 1.8 mm or 2.2 mm incisions [84]. Similar findings were reported by Yu et al. for 3.0 mm incisions versus 1.8 mm or 2.0 mm incisions [85].

In the included studies, some surgeries used incisions at the steep meridian [32,33], others used temporal incisions [35,37], and some did not specify the incision location [34,36,38,39]. This is relevant because corneal incisions along the steep meridian induce greater astigmatism reduction compared with off-axis incisions [50]. Furthermore, the studies did not specify whether mean or centroid SIA_Cornea_ values were used for tIOL calculations. The centroid value accounts for both magnitude and axis and is usually smaller than the mean, which reflects magnitude alone, and is considered more accurate for tIOL planning [83,86].

Overall, both UDVA and CDVA improved following tIOL implantation. The effect of tIOL implantation on CDVA is smaller than on UDVA. CDVA changes are less relevant when assessing the effectiveness of refractive surgery, as they would predominantly be the result of cataract removal [87]. Improvements in UDVA can be further attributed to the correction of sphere and cylinder with tIOL. In some studies, such as that by Abou Samra et al. and Jaimes et al., all but one KC patient underwent clear lens exchange [32,36]. In other words, it would be reasonable to postulate that the suboptimal visual quality of these patients was primarily due to optical aberrations rather than media opacity, assuming an absence of ocular comorbidities. Thus, one would expect such refractive surgeries to lead to greater improvements in UDVA than in CDVA postoperatively.

Increased magnitudes of HOAs are negatively correlated with corrected visual acuity [88]. Whilst lenticular opacities contribute to overall ocular HOAs, those in KC are mainly due to the air–tear interface at the anterior corneal surface [89,90,91]. Modern phacoemulsification surgery itself generally does not alter keratometric properties in a way that is beneficial in reducing corneal HOAs. In fact, corneal HOAs can increase due to corneal incisions during cataract surgery [88,92]. This review focuses on tIOLs, which cannot fully correct the aberrations present in keratoconic corneas. In contrast, customised aberration-correcting IOLs have been shown to reduce HOAs in eyes with non-progressive keratoconus [93]. Future studies comparing the refractive outcomes of tIOLs and customised IOLs in KC may provide further insights.

Four studies reported postoperative tIOL rotation. All used either AcrySof IQ tIOLs or Acrysof toric SN60TT IOLs, except for two cases in the series by Allard & Zetterberg [34,35,36,37]. In a randomised–controlled trial, Holland et al. demonstrated good rotational stability of AcrySof tIOLs. At one year, the authors reported a mean rotation of less than 4 degrees, with a range between 0 and 20 degrees [94]. Apart from one case of tIOL rotation requiring realignment surgery described by Jaimes et al. [36], no study reported any tIOL rotation of more than 10 degrees. Tognetto et al. concluded that tIOL rotation of greater than 10 degrees is significant for image quality loss [95]. This is consistent with the findings of Filipe et al. [96], who concluded that tIOL rotation of less than 10 degrees still provides satisfactory astigmatism correction. KC is associated with longer axial lengths on average, mainly due to longer posterior segments [97]. In turn, axial length is positively correlated with tIOL rotation [98]. However, no current studies have demonstrated an increased risk of tIOL rotation in keratoconic eyes.

The follow-up period in the included studies ranged from 1 to 31 months, including the case series by Ling et al., which reported an average follow-up of over 2 years. Five of the eight included studies had a follow-up of one year or less, highlighting a lack of long-term outcomes for tIOL implantation in KC. Whilst most cases in the included studies involved mild or moderate KC, different severity grading systems were used. The Amsler–Krumeich classification was cited in three studies [32,35,36]. Other staging systems referenced include the steepest K-value classification proposed by Thebpatiphat et al. [69] and a Pentacam-based system described by Hashemi et al. [99]. At present, no global consensus exists on KC classification [100], making subgroup analysis by severity unfeasible in this meta-analysis. Going forward, establishing a new consensus would improve the evidence base and better guide management.

In this review, forme fruste KC cases were excluded due to the lack of unified diagnostic criteria [101]. Another limitation of this review is the small number of eligible studies, reflecting the paucity of published data on this subject. Furthermore, all studies had small sample sizes, limiting statistical power. Finally, it is worth pointing out again that some studies did not specify their refraction method, and one case series partially reported refractive outcomes based on autorefraction, which is unreliable in KC [34]. This represents a notable weakness of the current literature.

## 5. Conclusions

tIOL implantation in KC appears to be a safe intervention for reducing astigmatism, based on a small number of heterogeneous studies. However, the true effectiveness of tIOLs in KC remains unclear due to the lack of vector analysis of astigmatic outcomes in the published literature. Moving forward, adopting standardised terminology, vector analysis methodology, and consistent reporting of astigmatic outcomes would help advance understanding and improve the effectiveness of tIOL implantation in KC.

## Figures and Tables

**Table 1:** Summary characteristics of included studies.

Study	Study Design	Country	Follow-Up	Preoperative Astigmatism	Sample Size	tIOL Model
Abou Samra WA et al., 2016 [32]	Prospective; CXL then tIOL after 6/12	Egypt	12 months	Mean: 4.53 ± 0.98 D	6 patients (9 eyes)	TECNIS toric IOL
Alió JL et al., 2014 [33]	Retrospective	Spain	3–15 months	Mean: −2.95 ± 1.71 D	10 patients (17 eyes)	Acrysof IQ toric IOL
Allard K et al., 2018 [34]	Prospective case series	Sweden	1–7 months	Mean: 6.94 ± 1.95 D	4 patients (4 eyes)	AcrySof IQ toric SN6AT IOL (patient 1 and 2), AT Torbi 709M(P) IOL (patient 3 and 4)
Hashemi H et al., 2015 [35]	Case series	Iran	3 months	Mean: 3.56 ± 1.84 D	17 patients (23 eyes)	AcrySof toric SN60TT IOL
Jaimes M et al., 2011 [36]	Retrospective	Mexico	3–31 months	Mean: 3.79 ± 1.88 D	6 patients (7 eyes)	AcrySof toric SN60TT IOL
Kamiya K et al., 2015 [37]	Prospective	Japan	3 months	Mean: 1.92 ± 1.73 D	19 patients (19 eyes)	AcrySof IQ toric SN6AT IOL
Ling JYM et al., 2023 [38]	Case series	Canada	Avg ≥ 2 years	Mean: 4.13 ± 2.25 D	17 patients (24 eyes)	Multiple models; unspecified
Ton Y et al., 2021 [39]	Case series	Israel	1 month	Mean: 2.95 ± 2.10 D	23 patients (32 eyes)	AcrySof toric SA6AT IOL, TECNIS toric IOL (ZCT series), Medicontur Bi-Flex toric IOL, Rayner T-flex toric IOL

## Data Availability

Data available upon written request to the corresponding author.

## Supplementary Materials

The following supporting information can be downloaded at https://www.mdpi.com/article/10.3390/life15091362/s1, Figure S1: PRISMA flow diagram; Table S1: MINORS risk of bias tool; Table S2: JBI critical appraisal checklist for case series.

## Abbreviations

The following abbreviations are used in this manuscript:

KC	Keratoconus
tIOL	Toric intraocular lens
SE	Spherical equivalent
UDVA	Uncorrected distance vision
CDVA	Corrected distance vision
REML	Restricted maximum likelihood
HOAs	Higher-order aberrations
RLE	Refractive lens exchange
SD	Standard deviation
D	Dioptre
MD	Mean difference
CMO	Cystoid macular oedema
CXL	Corneal cross-linking
TIA	Target induced astigmatism
SIA	Surgically induced astigmatism
DV	Difference vector
CI	Correction index
DAVD	Double-angle vector diagram
OCT	Optical coherence tomography
RA	Refractive astigmatism
KA	Keratometric astigmatism
LA	Lenticular astigmatism
PCA	Posterior corneal astigmatism
TCA	Total corneal astigmatism
IOL	Intraocular lens
