# Supplementary material for: Refractive Outcomes in Keratoconus Patients Following Toric Lens Implantation: A Systematic Review and Single-Group Meta-Analysis
Source: Life (Basel). 2025 Aug 27;15(9):1362. doi: 10.3390/life15091362 (PMC12471562; doi:10.3390/life15091362)

PRISMA 2020 flow diagram for new systematic reviews which included searches of databases and registers only

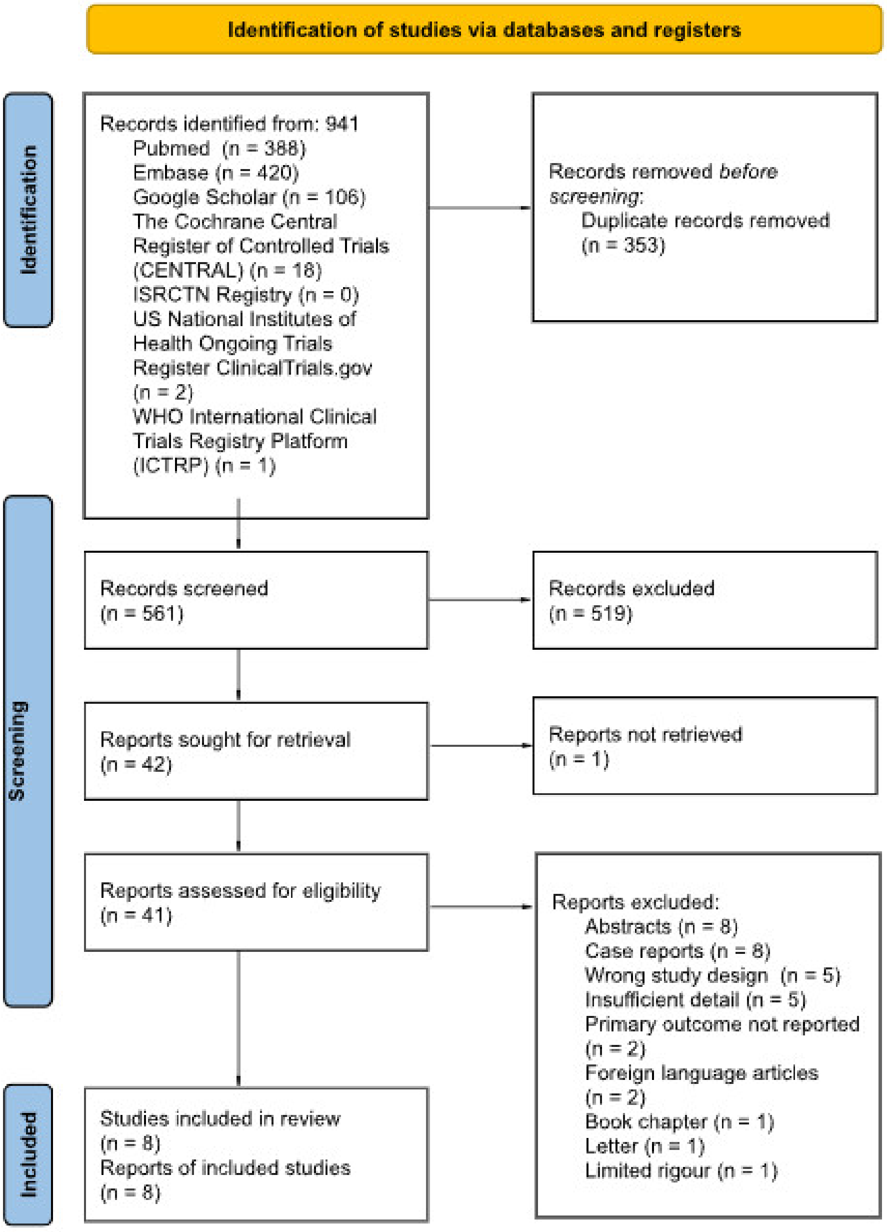

Supplement: Supplementary file 1 [file life-15-01362-s001.zip › Figure S1. PRISMA flow diagram.pdf]
