# Supplementary material for: Refractive Outcomes in Keratoconus Patients Following Toric Lens Implantation: A Systematic Review and Single-Group Meta-Analysis
Source: Life (Basel). 2025 Aug 27;15(9):1362. doi: 10.3390/life15091362 (PMC12471562; doi:10.3390/life15091362)
Supplement: Supplementary file 1 [file life-15-01362-s001.zip › Supplemental table S1 - MINORS risk of bias tool.pdf]

Supplementary Table S1: MINORS risk of bias tool

	A clearly stated aim	Inclusion of consecutive patients	Prospective collection of data	Endpoints appropriate to the aim of the study	Unbiased assessment of the study endpoint	Follow-up period appropriate to the aim of the study	Loss of follow-up less than 5%	Prospective calculation of the study size	Total score
Alió *et al.* 2014 [33]	2	0	1	2	1	1	2	0	9
Jaimes *et al*. 2011 [36]	1	1	1	2	0	1	2	0	8
Kamiya *et al.* 2016 [37]	2	2	2	2	1	2	2	0	11
Abou Samra *et al*. 2018 [32]	2	0	2	2	1	2	2	0	11
