# Supplementary material for: Refractive Outcomes in Keratoconus Patients Following Toric Lens Implantation: A Systematic Review and Single-Group Meta-Analysis
Source: Life (Basel). 2025 Aug 27;15(9):1362. doi: 10.3390/life15091362 (PMC12471562; doi:10.3390/life15091362)
Supplement: Supplementary file 1 [file life-15-01362-s001.zip › Supplemental table S2 - JBI critical appraisal checklist for case series.pdf]

Supplementary Table S2: JBI critical appraisal checklist for case series

	Were there clear criteria for inclusion in the case series?	Was the condition measured in a standard, reliable way for all participants included in the case series?	Were valid methods used for identification of the condition for all participants included in the case series?	Did the case series have consecutive inclusion of participants?	Did the case series have complete inclusion of participants?	Was there clear reporting of the demographics of the participants in the study?	Was there clear reporting of clinical information of the participants?	Were the outcomes or follow-up results of cases clearly reported?	Was there clear reporting of the presenting site(s)/clinic(s) demographic information?	Was statistical analysis appropriate?
Allard K & Zetterberg M 2018 [34]	Yes	Yes	Yes	Unclear	Unclear	Yes	Yes	Yes	N/A	No
Hashemi H et al. 2015 [35]	Yes	Yes	Yes	Unclear	Unclear	Yes	Yes	Yes	N/A	No
Ling JYM et al. 2023 [38]	Yes	Yes	Yes	Yes	Yes	Yes	Yes	Yes	N/A	No
Ton Y et al. [39]	Yes	Yes	Yes	Yes	Yes	Yes	Yes	Yes	N/A	No
